# Submandibular and sublingual salivary gland involvement in adenoid cystic carcinoma

**DOI:** 10.4322/acr.2024.500

**Published:** 2024-07-02

**Authors:** Poonam Ramnath Sawant, Manjeeta Mahesh Sinai Dhume, Anita Spadigam, Anita Dhupar

**Affiliations:** 1 Goa Dental College & Hospital, Department of Oral and Maxillofacial Pathology, Bambolim, Goa, India.

**Keywords:** Carcinoma, Adenoid Cystic, Salivary Gland Diseases, Sublingual Gland, Submandibular Gland

## Abstract

Adenoid cystic carcinoma (AdCC) is a malignant salivary gland neoplasm that presents as an indolent but aggressive neoplasm. AdCC histogenesis is linked to the intercalated ducts of the salivary glands, equally affecting the major and minor glands. AdCC is associated with distant metastasis, most commonly to the lungs, and a high recurrence rate. AdCC accounts for 4.2% of all tumors. About 55% of all reported cases affect the submandibular gland, and around 50% of AdCC cases occur in the minor salivary glands. The present review describes a case of AdCC which presented a single nodular swelling on the right side involving the floor of the mouth. It also consolidates the histopathological profile of a case of AdCC with all the relevant histopathological features.

## INTRODUCTION

Malignant salivary gland tumors account for 6-8% of all head and neck malignancies.^1^ Most parotid gland tumors are benign, while the sublingual gland tumors are usually malignant,^2^ and the submandibular gland shows an equal proportion of benign and malignant tumors.^3^ AdCC accounts for 4.2% of all tumors and represents 18.6% of all malignant tumors of the salivary glands.^1^ The parotid gland is the most often affected site, followed by the submandibular glands, which account for 55% of all the reported cases of AdCC. Approximately 50% of AdCC cases occur in the minor salivary glands, commonly affecting the hard palate.^4^ Minor salivary gland tumors are more frequently malignant than major salivary glands which are usually benign. The WHO has defined AdCC as a basaloid tumor containing epithelial and myoepithelial cells in diverse morphological configurations, such as tubular, cribriform, and solid patterns^5,6^. Its clinical course is relentless and usually has a poor prognosis. The present review consolidates the histopathological profile of a case of AdCC with all the relevant histopathological features. These include all three patterns of tumor cell proliferation, perineural, perivascular, intramuscular invasions, hyalinization of the stroma, focal areas of dedifferentiation. We also include a literature search.

## METHODS

**Figure 1:**
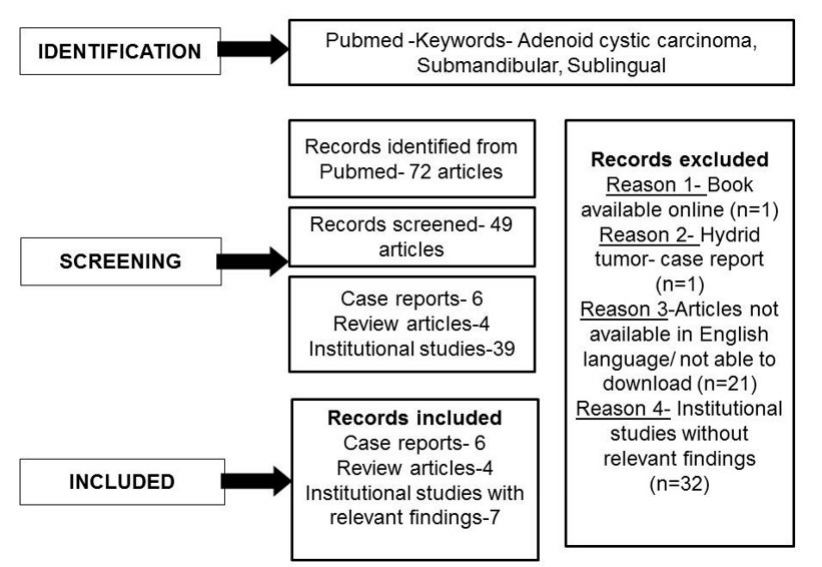
Flow chart showing segregation of the literature review.

An updated review of English language literature was performed using keywords –adenoid cystic carcinoma, submandibular, and sublingual, in the PubMed database, revealed seventy-two articles - comprising original research, case series, case reports, and reviews. Our literature review is presented in the Figure 1.

## RESULTS

Literature obtained from the Pubmed database yielded six case reports, four review articles, and seven institutional studies. Relevant data pertaining to demographics and clinico-histopathological findings were analyzed from these articles.

Six case reports were analyzed and summarized in Table 1. AdCC was commonly reported on the floor of the mouth. The cases reported by Whear and Addy,^8^ McFall et al.^9^ and Saito et al.^10^ and Kumar et al.,^12^ mentioned that the AdCC probably originated from the sublingual salivary gland. Histologically, the cribriform pattern was seen in all cases. Only one case report distinctly reported perineural and perivascular invasion.

**Table 1:** Details of 6 AdCC cases and the present case

Ref.	Age(y)/Gender	Site	Histopathology			
			Pattern	PN	PV	Other
^7^	58/F	Right SM region	Cribriform	-	-	-
^8^	57/F	Left FOM	Cribriform (Sublingual)	-	-	-
^9^	16/F	Right FOM	Cribriform +Tubular (Sublingual)	+	+	-
^10^	73/M	Right FOM	Cribriform + Solid (Sublingual)	-	-	-
^11^	43/F	Left SL region	Cribriform	-	-	-
^12^	64/M	Right FOM	Cribriform (Sublingual)	-	-	-
PC	59/M	Right FOM	Cribriform + Tubular + Solid (Submandibular + Sublingual)	+ SH-HGT	+ SH-HGT	+ muscle

FOM= floor of mouth; PC= present case; PN= perineural; PV= perivascular; Ref = reference, SH-HGT= stroma hyalinization and high-grade transformation; SL= sublingual; SM= submandibular.

## CASE REPORT

**Figure 2:**
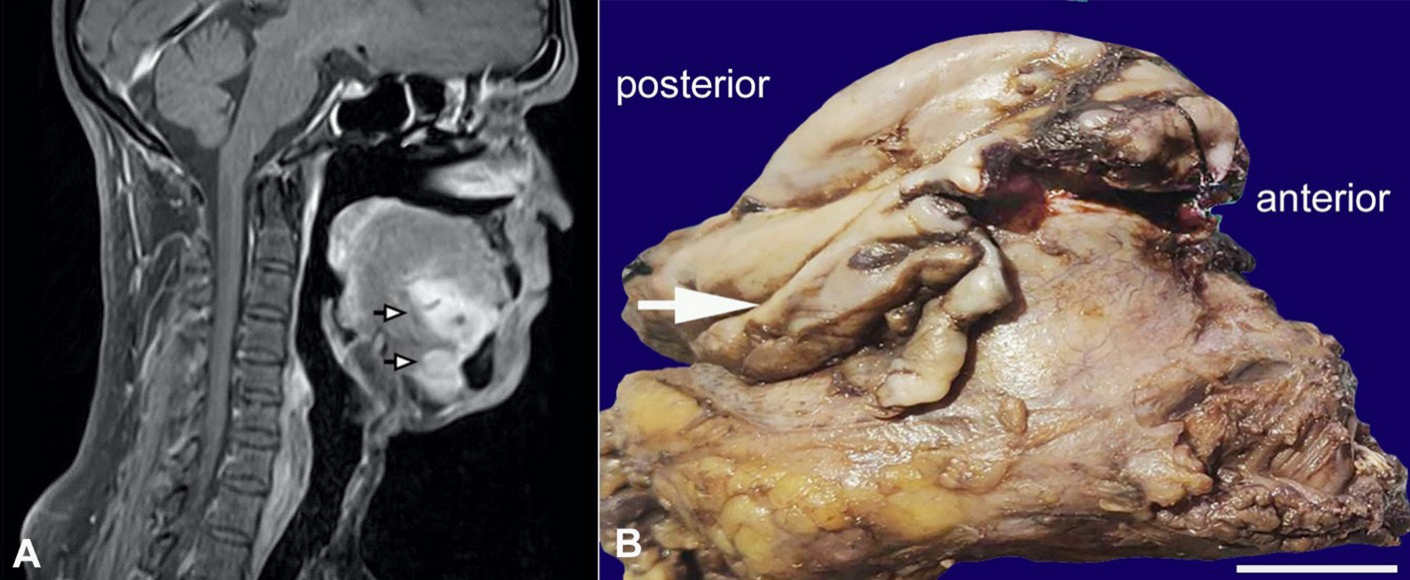
**A –** MRI of the right side of the neck (axial view) lesional area pointed with black&white arrowheads. Lesion appears heterogeneously hyperintense on T2 and STIR sequences and isointense on T1Wi; **B –** Gross view of the surgical specimen showing a single mass without distinction between sublingual and submandibular gland (white arrow) (scale bar= 1,5 cm).

A fifty-nine year old male patient was referred to the department to evaluate a swelling in the floor of the mouth. On intra-oral examination, a single nodular swelling was noted on the right side of the floor of the mouth, with intact overlying mucosa measuring approximately 4x2cm. Right submandibular lymph nodes were palpable, tender, and mobile, measuring approximately 1.2x0.8cm. The magnetic resonance imaging (MRI) of the neck’s right side showed a large well-defined lesion involving the sublingual space, infiltrating into the mylohyoid muscle, abutting onto the anterior belly of digastric muscle. No invasion of the bone or tongue muscles was found (Figure 2A).

On gross examination, a single mass was found without distinction between sublingual and submandibular glands (Figure 2B).

An incisional lesion biopsy rendered a diagnosis of AdCC of the floor of the mouth.

**Figure 3:**
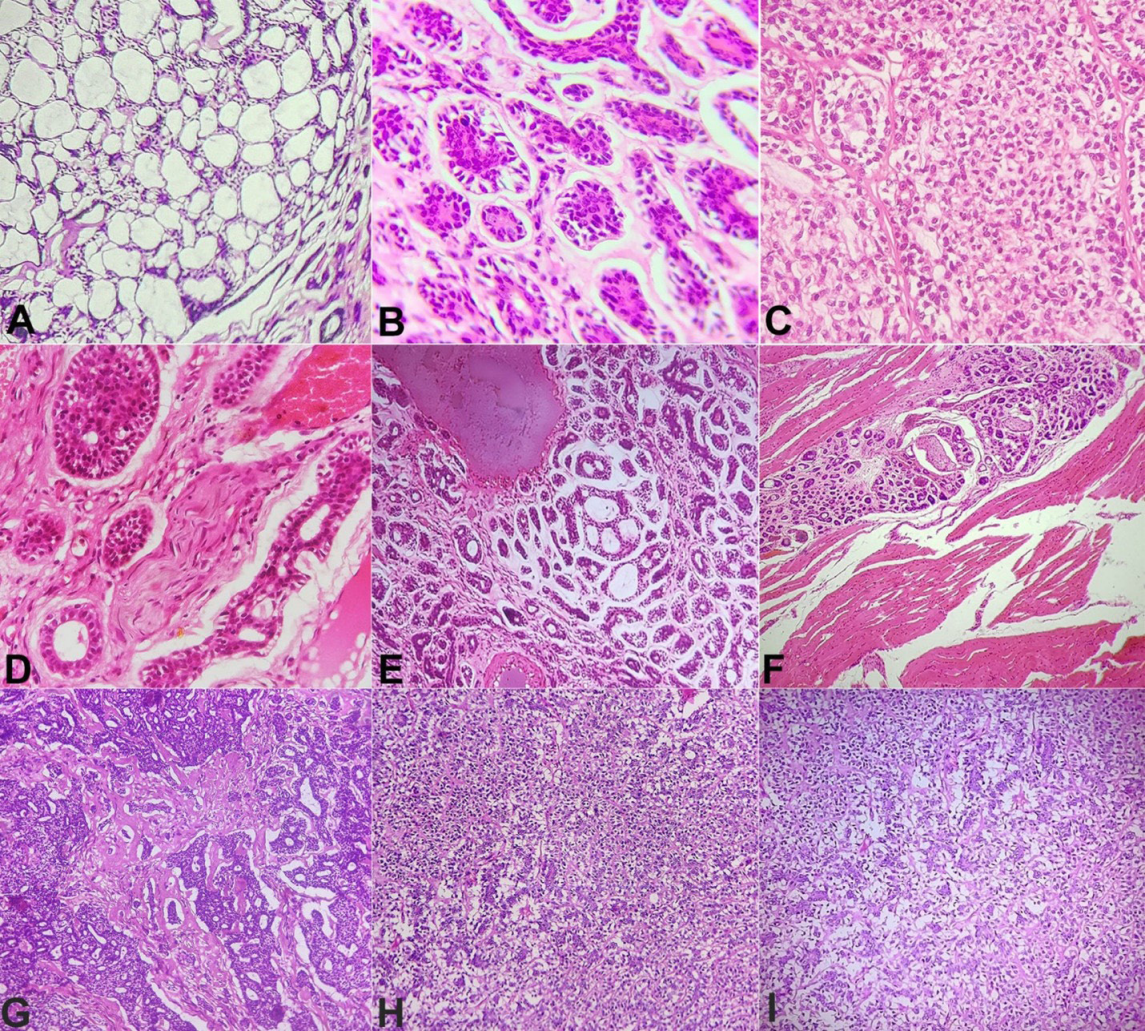
Photomicrograph of AdCC with distinct histopathologic features – **A –** Cribriform pattern; **B –** Tubular pattern; **C –** Solid pattern; **D –** Perineural invasion (Black arrow); **E –** Perivascular invasion (Red arrow); **F –** Intramuscular invasion; **G –** Hyalinization of stroma; **H –** areas of high-grade transformation; **I –** Few Clear cells in areas of high-grade transformation. (H&E, 100x).

Subsequently, the resected specimen of the right submandibular and sublingual salivary gland, along with the right side supra-omohyoid lymph node dissection, was received in the department. A final diagnosis of AdCC Grade II (Szanto)^13^ of sublingual and submandibular gland was concluded. All the margins and the lymph nodes were free of tumor cells. The present case of AdCC revealed some unique histopathological findings, as follows: (Figure 3): (i) Complete replacement of glandular tissue (submandibular and sublingual glands) by the tumor tissue; (ii) All three histological patterns i.e, Cribriform, tubular and solid were evident. (Figures 3A, 3B, and 3C); (iii) There was evidence of perineural [Classified as Extratumoral, peripheral as proposed by Miller et al.],^14^ perivascular and intramuscular invasion, along with areas of hyalinization in the connective tissue stroma. (Figures 3D, 3E, and 3F); (iv) The tumor core showed few areas of high-grade transformation and focal areas with clear cells. (Figures 3G, 3H, and 3I).

Seven studies^15-21^ made a distinct mention of perineural, perivascular and intramuscular invasion were analyzed and their findings were summarized in Table 2. This review showed that in addition to perineural invasion, other features like the status of tumor margin, clinical tumor stage, nerve and lymph node involvement, are crucial in determining prognosis in patient's with AdCC.

**Table 2:** Seven studies concerned with perineural, perivascular, and intramuscular invasion

Invasion	Reference/ n cases	Prognosis
Perineural invasion	^15^/n=60	Margin status and perineural invasion predict disease-free survival or overall survival
	^16^/n=59	Although clinical T stage, gross nerve invasion, and nodal involvement before treatment were adverse prognostic factors, routine radiation of the skull base may reduce the bad prognostic significance of the perineural invasion
	^17^/n=33	Perineural invasion did not demonstrate a statistically significant association with survival.
	^18^/n=198	Perineural invasion was an adverse prognostic factor only when a major nerve was involved.
	^19^/n=30	Need to do a frozen section examination of the peripheral margins along with the adjacent nerve that is most likely involved.
	^22^ and ^20,^	perineural or clinically named nerve invasion alarms for a worse prognosis
	^21^	Perineural involvement does not impact outcome.
Perivascular invasion	^17^/n=33	5 cases of Perivascular invasion (No mention of its significance)
Other tissue invasion	^17^/n=33	15 cases of muscle or tissue invasion (No mention of its significance)

## DISCUSSION

AdCC differs from other malignant salivary gland neoplasms in its slow but relentless growth, its tendency towards local recurrence, and a paradoxically high five year but dismally low 10-20 year survival rate. The distant metastasis is to the lungs via a hematogenous route.

AdCC shows almost an equal distribution amongst major and minor salivary glands.^4^ Although this tumor is likely to occur at almost any age, it is most commonly observed in women in the fifth and sixth decades of life.^23^

Salivary gland neoplasms usually present as a solitary mass involving a single gland. Neoplastic involvement of multiple glands or adjacent glands concomitantly is unusual. Seifert and Donath^24^ used distinct terminology to describe the simultaneous neoplastic involvement of multiple salivary glands. They addressed the following three perspectives: (i) histologic type i.e., neoplasm with identical or different histology, (ii) time of development i.e., synchronous or metachronous development of the neoplasm, and (iii) localization of the neoplasm i.e., unilateral or bilateral. Our case showed histopathologically identical neoplastic tissue involving both the sublingual and the submandibular glands, causing complete effacement of the salivary gland architecture and discontinuity of the glandular capsule. The findings above indicate a probable synchronous AdCC development involving both the sublingual and submandibular salivary glands. However, whether the AdCC was a collision tumor, i.e., two malignant tumors occurring at independent sites (multifocal), which subsequently meet or collide as the tumor spreads, or a unifocal tumor involving the sublingual gland locally and then extending to involve the adjacent submandibular gland is questionable.

In our case, the complete effacement of both salivary glands challenged the determination of the AdCC origin; however, reviewing the literature, we found four cases in which the sublingual salivary gland was concluded to be the gland of origin. (Table 1) This may be attributed to unique anatomical features of the sublingual gland, i.e., it lacks a distinct gland capsule, it is drained by multiple excretory ducts that are in close proximity to the submandibular duct, and the postganglionic lingual nerve traverses to the anterior portion of the tongue through or near the sublingual glands. Hence, tumors arising from the sublingual glands have a high probability for invasion into the neighboring structures, which necessitates excision of the submandibular gland during surgical resection of malignant sublingual gland tumor.^25^

The AdCC tends to invade the adjacent nerve sheaths close to the primary tumor and spread along the nerve. Most of the authors as mentioned in Table 2^15-17,20,22^ reported that perineural or neural invasion is usually indicative of a worse prognosis. Gurney et al.^17^ and Sur et al.^21^ did not find any statistical significance between perineural invasion and overall patient survival.

Interestingly, only Gurney et al.^17^ documented perivascular and intramuscular invasion of AdCC, along with perineural invasion, however, its significance in the prognostication of AdCC is yet to be established.^26,27^

AdCC is a biphasic neoplasm composed of ducts and abluminal myoepithelial cells arranged in tubular, cribriform, and solid growth patterns embedded in an acellular stroma.^28^ In the present case, all 3 growth patterns were noted along with distinct hyalinization of the stroma and focal areas of high-grade transformation. Hyalinization of the stroma^29^ and high-grade transformation of AdCC represent oncological events associated with the aggressive nature of the neoplasm and adverse clinical outcome.^30^

Today, the various histopathological grading systems proposed in the literature consider the tumor histopathological pattern and the evident percentage of solid growth pattern, compared to the tubular or cribriform pattern seen in neoplastic tissue (Table 3).^5,13,21,31,34^

**Table 3:** Various grading systems for AdCC

Grading systems	Histopathological Grades of AdCC		
	Grade I	Grade II	Grade III
Spiro and Huvos^31^	Mostly Tubular or Cribriform, Occasionally Solid	Mixed with substantial Solid	Only solid
Perzin et al.^32^/ Szanto et al.^13^	Predominantly Tubular, No solid	Predominantly Cribriform, <30% Solid	Solid component >30%
John and Chan^33^	Tubular and cribriform together, without a solid pattern	Mostly cribriform, with less than 30% of solid pattern	Solid being the predominant subtype
van Weert et al.^34^	No Solid component	Presence of Solid component (Any %)	-
Morita et al.^5^ Min A max-Minor Axis maximum	Min A max < 0.20 mm	Min A max >0.20 mm	-

The present case of AdCC showed < 30% of solid growth pattern; hence, it was assigned as grade II. Although tumor microenvironment and host factors such as lympho-vascular invasion, perineural invasion, and stromal hyalinization are independently considered important prognostic indicators, the current grading systems for AdCC do not consider them unlike mucoepidermoid carcinoma.

## CONCLUSION

AdCC of sublingual and submandibular salivary glands is rare. The present case could be the first documented case of AdCC involving both the sublingual and submandibular salivary glands and with distinct histopathological findings. A thorough review of the literature and evaluation of the present case highlighted the need to have a more consolidated grading system for AdCC, which would consider all important histopathological prognosticators such as perineural, perivascular and intramuscular invasion, along with hyalinization of connective tissue stroma and the high-grade transformation. In the present case, the patient was treated with surgical resection, and at one year and ten months follow-up showed no recurrence.
